# Chlorambucil Monotherapy in Dogs with Protein-Losing Nephropathy of Probable Immune Origin: A Preliminary Study

**DOI:** 10.3390/vetsci12080721

**Published:** 2025-07-31

**Authors:** Felipe Díaz-Soler, María José Bernal, Franco Gonzalez, Ismael Pereira, Francisco Abusleme, Daniela Siel

**Affiliations:** 1 Escuela de Medicina Veterinaria, Facultad de Medicina y Ciencias de la Salud, Universidad Mayor, Santiago 8580745, Chile; franco.gonzalezv@umayor.cl (F.G.); ismael.pereira@umayor.cl (I.P.); francisco.abusleme@umayor.cl (F.A.); 2 Escuela de Medicina Veterinaria, Facultad de ciencias de la Vida, Universidad Andrés Bello, Santiago 8370251, Chile; maria.bernal@unab.cl; 3 Centro de Biomedicina, Universidad Mayor, Santiago 8580745, Chile

**Keywords:** protein-losing nephropathy, dogs, immune complex-mediated glomerulonephritis, chlorambucil, kidney disease

## Abstract

**Simple Summary:**

Protein-losing nephropathy secondary to immune-complex-mediated glomerulonephritis (ICGN) in dogs is a severe condition characterized by immune-mediated damage of the glomerular filtration barrier leading to persistent proteinuria and renal injury. Treatment of ICGN requires immunosuppressive therapy which usually includes drugs such as prednisolone, mycophenolate, cyclophosphamide. However, there are no reports of the use of chlorambucil as immunosuppressive monotherapy. This study describes five clinical cases in which chlorambucil was used as the sole immunosuppressant agent in dogs with protein-losing nephropathy (PLN) of probable immune-mediated origin, with favorable clinical and paraclinical results.

**Abstract:**

Protein-losing nephropathy (PLN) involves a heterogeneous group of pathologies leading to selective glomerular damage and development of renal disease. ICGN, the main cause of PLN, requires immunosuppressive treatment. However, the scientific evidence in veterinary medicine on immunosuppressive therapeutic schemes in this condition is limited. The aim of this study is to describe the clinical and paraclinical evolution of five dogs with PLN, presumably associated with ICGN, treated with chlorambucil as immunosuppressive monotherapy. Suspected IGCN was established by the presence of a urine protein–creatinine ratio (UPC) ≥ 3 without response to standard therapy, hypoalbuminemia < 2, or progressive azotemia. Patients were treated with a dosage range of chlorambucil from 0.16 to 0.4 mg/kg (mean 0.25 mg/kg) every 24 h as the sole immunosuppressant. In the end, 4/5 patients showed significant clinical improvement, 3/3 had resolution of the nephrotic syndrome, 5/5 had a sustained decrease in UPC values during follow-up and no relevant adverse effects were observed. In this report, chlorambucil proved to be a well-tolerated and potentially effective monotherapy for immune-mediated PLN in dogs.

## 1. Introduction

Chronic kidney disease (CKD) is a prevalent condition in dogs, with a reported prevalence ranging from 0.5% to 3.7%. This disease not only compromises life expectancy but also significantly affects the quality of life of affected individuals [1,2,3]. The presence of proteinuria, characterized by an elevated urine protein-to-creatinine ratio (UPC) over time, is associated with a poorer prognosis [4,5]. On the other hand, sustained and severe proteinuria is a marker of glomerular disease pathology that encompasses a heterogeneous group of disorders that selectively affect the glomeruli and can originate as a result of systemic inflammatory, neoplastic, metabolic, or genetic diseases, leading to renal injury and fibrosis. Indeed, among the different causes of CKD in dogs, protein-losing nephropathy (PLN) is increasingly recognized as a significant contributor; although its precise prevalence remains unknown, glomerular disease is highly prevalent among patients with established CKD [6].

**Figure 1:**
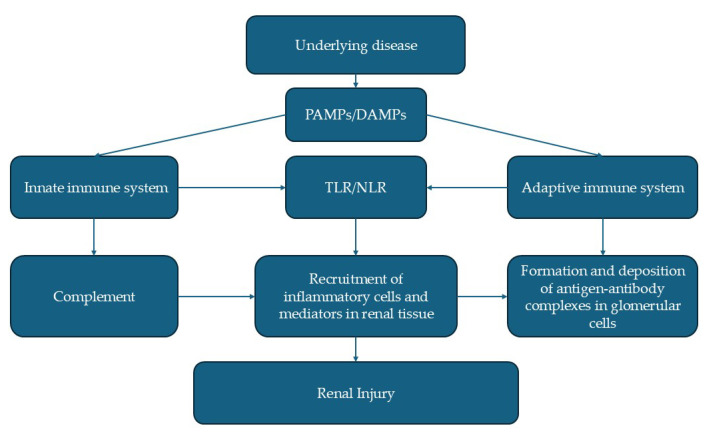
Schematic representation of immune-mediated mechanisms leading to renal glomerular damage.

In dogs, immune-complex mediated glomerulonephritis (ICGN) is the most frequently reported cause of PLN, with a prevalence ranging from 30% to 50%, depending on the geographical region and the animal population studied dogs and cats [7,8,9]. In recent studies, membranoproliferative glomerulonephritis is among the most commonly identified pathologies in biopsies of dogs with established chronic kidney disease, which has been associated with sulphonamide administration in Doberman dogs, with neoplasia, chronic inflammatory processes, and infectious diseases such as chronic bacterial (brucellosis, mycoplasmosis) and vector diseases (haemobartonel-losis, leishmaniosis, borreliosis, babesiosis, ehrlichiosis, dirofilariasis) [10,11]. According to the most recent studies, glomerular diseases, particularly membranoproliferative glomerulonephritis, are among the most commonly identified pathologies in biopsies of dogs with established chronic kidney disease. However, membranoproliferative glomerulonephritis has been associated with a better prognosis and longer survival, which also depends on serum creatinine values, UPC values, and serum albumin concentrations. [10] The pathogenesis of this disease relies on immune responses triggered by pathogen-associated molecular patterns (PAMPs) or damage-associated molecular patterns (DAMPs), which activate both the innate and adaptive immune systems. The innate immune system responds immediately through Toll-like receptors (TLRs) and Nod-like receptors (NLRs) expressed on inflammatory and resident glomerular cells, releasing inflammatory mediators that damage the glomeruli. Additionally, PAMPs and DAMPs activate the complement pathway, attracting circulating inflammatory cells and stimulating resident glomerular cells. The adaptive immune system promotes antibody production, leading to the deposition of circulating or in situ–formed immune complexes. These complexes can further activate TLRs via alternative pathways, amplifying glomerular injury (Figure 1) [12].

Despite the central role of immunosuppression in managing ICGN, there is a critical lack of clinical data supporting specific therapeutic protocols in dogs.

The clinical manifestations of PLN vary widely and depend on the degree of proteinuria, the IRIS stage of renal disease, and the presence of clinical syndromes or complications associated with proteinuria [13]. Early diagnosis and timely treatment of primary glomerular diseases are critical to reducing renal injury and preventing the progression to chronic kidney disease.

The definitive diagnosis of PLN requires histopathologic evaluation of renal cortical biopsy samples using light microscopy, transmission electron microscopy, and immunofluorescence [14]. The current standard treatment for PLN in dogs focuses on reducing proteinuria by inhibiting the renin-angiotensin-aldosterone system (RAAS), primarily through the use of angiotensin-converting enzyme inhibitors (ACEIs) or angiotensin II receptor blockers (ARBs) [15]. However, there is evidence suggesting that the use of ARBs may be theoretically more advantageous, as they selectively inhibit angiotensin II type 1 receptors, thereby preserving the beneficial effects mediated by type 2 receptor activation and avoiding ACE-independent proteolytic pathways [16]. This approach helps decrease intraglomerular pressure and mitigates progressive damage to the glomerular filtration barrier. However, in cases with histopathologic confirmation of immune-complex glomerulonephritis (ICGN), it is essential to identify and eliminate the underlying disease that promotes the activation of the innate immune system, the deposition of antigen-antibody complexes, and the sequestration of inflammatory mediators within the glomerular tissue. Additionally, immunosuppressive therapy is recommended to modulate the aberrant immune response and delay the progression of glomerular damage, especially when the cause of ICGN is unknown or unavoidable. In clinical scenarios where renal biopsy is contraindicated or not feasible due to technical, economic, or medical limitations, empirical immunosuppressive therapy may be considered. Such decisions should be based on the patient’s clinical course, particularly in dogs with progressive azotemia or marked hypoalbuminemia despite standard therapy with ACEIs or ARBs, especially when serum albumin levels fall below 2.0 g/dL [17].

Due to the limited evidence available on the effectiveness of immunosuppressive therapy in immunocomplex-mediated glomerular disease, treatment guidelines in dogs are based on recommendations of expert opinion established in the IRIS proteinuria consensus. These guidelines propose the potential clinical benefits of various immunosuppressive agents in slowing disease progression, reducing severity, and improving outcomes. To date, mycophenolate mofetil, either as monotherapy or in combination therapy, has been the most recommended and utilized immunosuppressant for this condition [18,19].

Nevertheless, alkylating agents such as chlorambucil may represent a safe alternative for the management of immune-mediated PLN. Chlorambucil is a chemotherapeutic agent widely used in the treatment of leukemia and lymphoma and has also been employed in certain immune-mediated diseases, particularly in feline patients and in mesangioproliferative glomerulonephritis (MPGN) in humans. Alkylating agents form covalent bonds with DNA, inducing cross-linking between DNA strands, which triggers apoptosis in rapidly proliferating cells, particularly B and T lymphocytes [20]. The reduction of antigen-antibody complexes deposited in the glomerular tissue and the consequent decrease in the activation of the complement system modulate the local aberrant immune response, which may lead to decreased proliferation of mesangial and endothelial cells at the glomerular level, characteristic features of MPGN [21]. In this regard, in humans, the combined use of glucocorticoids (GCS) with an alkylating agent such as cyclophosphamide in 84 patients with MPGN demonstrated that the combination of cyclophosphamide and GCS was more effective in achieving remission than GCS alone [22]. Adverse effects reported in dogs treated with chlorambucil are mild gastrointestinal symptoms, such as loss of appetite, vomiting, or diarrhea (39.5%). Hematological effects, such as thrombocytopenia and neutropenia, are considerably less common (13.2%) and are described as dose-dependent and reversible once established [23,24].

The aim of this preliminary study is to describe the clinical and laboratory response to chlorambucil monotherapy in five dogs with suspected immune-mediated PLN in order to explore its potential role as a safe and effective therapeutic alternative and contribute to the current body of veterinary evidence on glomerular disease management.

## 2. Materials and Methods

### 2.1. Study Population and Inclusion Criteria

Dogs diagnosed with protein-losing nephropathy (PLN) and treated with chlorambucil immunosuppressive monotherapy were included in the study. Dogs with PLN were identified by the presence of sustained and severe proteinuria. Severe proteinuria was defined as a sustained UPC ≥ 3. Patient selection criteria included an inadequate clinical and paraclinical response to standard glomerular therapy, characterized by a decrease in the protein–creatinine ratio (UPC) of less than 50%, urine UPC ≥ 3, persistent hypoalbuminemia (<2 mg/dL), and/or progressive azotemia.

Before initiating immunosuppressive treatment with chlorambucil, thoracic radiographs, abdominal ultrasound, serum biochemical profiles, complete blood counts, complete urinalysis with urinary sediment analysis, and UPC determinations were performed on all five dogs in the study. In addition, Borrelia, Anaplasma, Ehrlichia, and Dirofilaria infections were ruled out using the IDEXX SNAP 4Dx test.

Medical records of dogs treated with immunosuppressive chlorambucil monotherapy were examined, including age, sex, body weight, biochemical profile, complete blood count, complete urinalysis.

Medical follow-up of the dogs was performed every 15 days until an adequate clinical and paraclinical response was observed, characterized by a decrease in UPC values of at least 50%, as well as by a reduction of clinical signs, such as inappetence, abdominal effusion, or edema. Follow-up of dogs on treatment with chlorambucil was performed every three months. In non-azotemic patients who completed immunosuppressive therapy, follow-up was performed every six months, while in azotemic patients it was performed every three months during the entire follow-up period. Medical controls and records were performed by a single physician during the entire follow-up period. The follow-up time was 2800 days.

### 2.2. Standard Treatment for Glomerulopathy

Prior to the initiation of immunosuppressive therapy, all patients were managed according to the guidelines established by the consensus for the treatment of glomerular disease in dogs [15]. Standard therapy consisted of administration of a renin-angiotensin-aldosterone system inhibitor (enalapril) or an angiotensin receptor blocker (telmisartan) at doses of 0.5 to 1 mg/kg/day to control proteinuria. Antiproteinuric therapy was only discontinued in those dogs who presented an increase in serum creatinine concentration of more than 25% with respect to their baseline value throughout the treatment period and was only restarted once serum creatinine values remained stable. Aspirin at a dose of 1 mg/kg/day was administered as antithrombotic prophylaxis to patients with hypoalbuminemia lower than 2, together with a UPC higher than 3, and its administration was suspended in dogs with an increase in albumin higher than 2 with a decrease in UPC value lower than 3. Amlodipine was administered as an antihypertensive to those dogs with persistent systemic arterial hypertension (>160 mmHg). Additionally, all dogs received omega-3 fatty acid supplementation at a dose of 100 mg/kg/day.

### 2.3. Immunosuppression Protocol with Chlorambucil

All patients received chlorambucil administered orally, in a dosage range of 0.16 to 0.4 mg/kg (mean 0.25 mg/kg) once daily, following the recommendations established in the consensus for the management of glomerular diseases in dogs [18].

Immunosuppressive therapy was considered completed in those patients who met clinical and paraclinical stability criteria, defined as follows: sustained reduction of UPC to values < 0.5 or a reduction ≥ 50% from baseline, maintained in at least two consecutive measurements separated by a minimum of 30 days; stability in serum creatinine values in serial measurements; absence of clinical signs of hypoalbuminemia, such as cavitary effusions or dependent edema.

### 2.4. Statistical Analysis

Descriptive statistics were performed with R statistical using the software package Rcmdr and Survival for survival analysis (v4.5.0; R Core Team, 2021; Vienna, Austria), considering interest variables of age, serum creatinine, serum urea, serum phosphorus, UPC, serum albumin, nephrotic syndrome. For serum creatinine, serum phosphorus, and UPC values, cut-off values recommended by the International Renal Interest Society (IRIS) were used. Statistical significance was set at ≤0.05.

The data were tested for normality using the Shapiro–Wilk test and are presented as either the mean with standard deviation (SD) or the median with range, since no normality was detected and non-parametric tests were used.

The Kaplan–Meier test was performed for all dogs with each variable of interest. The log-rank test (Mantel–Cox) was used to analyze the presence of significant differences among survival curves.

## 3. Results

### 3.1. Characterization of Patients

Five dogs treated with chlorambucil immunosuppressive monotherapy were retrospectively enrolled between August 2017 and August 2022.

Three patients treated with immunosuppressive therapy were neutered males, and two were spayed females, which included 2 Cross breed dogs, 1 Brazilian Mastiff, 1 Maltese and 1 Cocker Spaniel. Age at diagnosis ranged from 5 to 12 years (mean 8 years). The five dogs included did not have updated medical records prior to the development of PLN, so it could not be established whether they had kidney disease prior to the diagnosis of PLN. One of the patients had a history of immune-mediated hemolytic anemia but without ongoing treatment.

UPC values prior to initiation of immunosuppressive therapy ranged from 6.5 to 13.2 mg/dL (mean 9.28). Three had albumin levels less than 2 mg/dL (mean 1.4 mg/dL) without azotemia, and two had progressive azotemia with hypoalbuminemia greater than 2 mg/dL (2.3 mg/dL). Three patients had nephrotic syndrome, two of them presented with abdominal effusion, and one exhibited abdominal effusion plus dependent edema and hind limb edema (Table 1).

Prior to the initiation of immunosuppressive therapy, one patient received enalapril at a dose of 0.5 mg/kg once daily for two weeks, followed by 0.5 mg/kg every 12 h for an additional two weeks. After immunosuppressive therapy was initiated, the enalapril dose was adjusted to 0.5 mg/kg once daily. Two patients were treated with telmisartan at a dose of 1 mg/kg once daily, with a mean treatment duration of 21 days prior to the start of chlorambucil therapy. Once immunosuppressive therapy commenced, the telmisartan dose remained unchanged. Two additional patients were treated with telmisartan at a dose of 0.5 mg/kg once daily. Both exhibited progressive azotemia before and after the initiation of telmisartan. The duration of telmisartan administration before starting immunosuppressive therapy was 12 days. In both cases, telmisartan was discontinued upon initiation of immunosuppressive therapy due to an increase in serum creatinine exceeding 25% of baseline. Once serum creatinine levels stabilized, telmisartan therapy was reinstated at 0.5 mg/kg/ once daily for the entire follow-up period. Prior to the initiation of immunosuppressive treatment, no patient demonstrated a reduction in UPC values.

One patient was maintained on treatment with amlodipine at a dose of 0.2 mg/kg once daily during the entire follow-up period. Three patients received aspirin at a dose of 1 mg/kg once daily, with a mean administration time of 35 days.

None of the patients had a history of recent vaccinations or travel within or outside the country.

All the patients presented hyporexia, weight loss, and a moderate decline prior to immunosuppressive treatment. Two patients presented tachypnea associated with the presence of severe abdominal effusion, a respiratory pattern that improved when abdominocentesis was performed. Analysis of the abdominal fluid was compatible with pure transudate.

None of the dogs included in the study presented thromboembolism during the entire follow-up period.

### 3.2. Outcome

Three patients completed immunosuppressive therapy with chlorambucil, one was euthanized 45 days after initiation of treatment, a decision adopted by the guardians due to unfavorable clinical evolution, and one patient continued treatment indefinitely. In patients who completed therapy, the duration of chlorambucil treatment ranged from 60 to 210 days, with a median of 110 days. Once the immunosuppressive therapy was completed, the frequency of administration of chlorambucil was extended to every 48 h for 3 weeks, and then the medication was discontinued in the absence of clinical recurrence.

At the end of the therapeutic protocol, two of the three patients who completed therapy were classified as having stage 1 chronic kidney disease (CKD), according to the International Renal Interest Society (IRIS) guidelines, while one was classified as stage 2. In the patient who maintained continuous immunosuppressive therapy, stage 2 CKD was determined after 60 days of treatment, given that serum creatinine values remained unchanged during that period.

**Figure 2:**
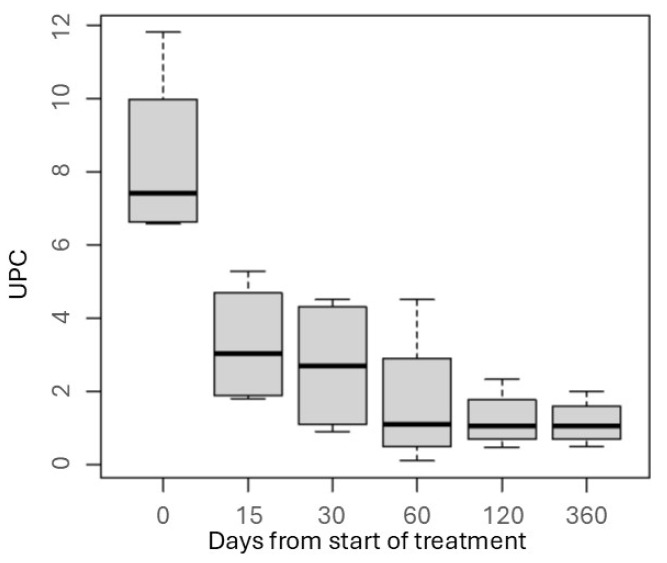
Boxplot showing the variation in urine protein-to-creatinine ratio (UPC) over time in dogs with suspected immune-mediated protein-losing nephropathy treated with chlorambucil as immunosuppressive monotherapy. Data are presented for five dogs; however, one dog died at day 45, and, therefore, no data were available for this patient at subsequent time points. For the remaining four dogs, UPC values were recorded for all scheduled sampling dates. The boxes represent the interquartile range (IQR), the horizontal line within each box indicates the median, and the whiskers represent the minimum and maximum values.

In all cases, a sustained reduction in the urine protein–creatinine ratio (UPC) was observed during treatment with chlorambucil. One patient showed a decrease in UPC of less than 0.5, while the remaining patients showed a reduction of more than 50% from baseline. The time required to achieve a greater than 50% reduction from baseline UPC ranged from 15 to 120 days, with a median of 1.2 after 120 days of treatment (Figure 2).

**Figure 3:**
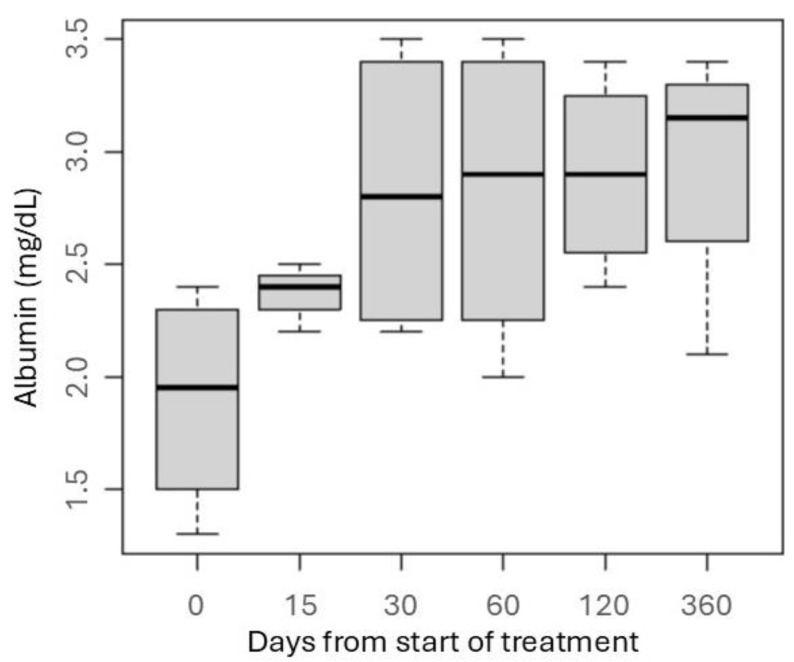
Boxplot showing changes in serum albumin concentration in response to chlorambucil monotherapy in dogs with protein-losing nephropathy of suspected immune-mediated origin. Data are presented for five dogs; however, one dog died at day 45, and, therefore, no data were available for this patient at subsequent time points. For the remaining four dogs, UPC values were recorded for all scheduled sampling dates. The boxes represent the interquartile range (IQR), the horizontal line within each box indicates the median, and the whiskers represent the minimum and maximum values.

Likewise, all patients experienced a progressive increase in serum albumin from 15 days after initiation of immunosuppressive treatment. Normalization of serum albumin levels was achieved in two patients at 30 days, in one at 120 days; only one patient maintained persistent mild hypoalbuminemia (Figure 3).

In relation to serum creatinine, at 15 days of treatment, two patients showed a percent increase of more than 50%, while one showed a reduction of 10.7%, and two showed increases of less than 17%. At 30 and 60 days, three patients presented negative percent variations in creatinine levels. Thus, three dogs presented a creatinine value with a variation ≤ 0.3 mg/dL with respect to the baseline value. However, one dog died after 45 days, so it was not possible to establish a long-term response to treatment, and two dogs presented a sustained decrease in creatinine values with respect to the baseline value after 15 and 30 days of treatment with chlorambucil (Figure S1a). Additionally, a percent increase in hematocrit values was documented in all patients after 60 days of treatment. On the other hand, a percent decrease from baseline in leukocyte and platelet counts was observed during 60 days of chlorambucil treatment (Table 2); however, no dog developed leukopenia or thrombocytopenia. Leukocyte counts ranged from 7830 to 12,100 Leu/µL (median 11,400 Leu/µL) and platelet counts ranged from 248,000 to 697,000 PLT/µL (median 316,000 PLT/µL) at day 60 of treatment (Figure S1b,c).

None of the patients treated with chlorambucil presented diarrhea, vomiting, leukopenia; however, one patient had persistent anorexia throughout the treatment with chlorbucyl. The three patients who presented with nephrotic syndrome with abdominal effusion or peripheral edema showed disappearance of abdominal effusion and edema 48 h after initiating immunosuppressive therapy.

**Figure 4:**
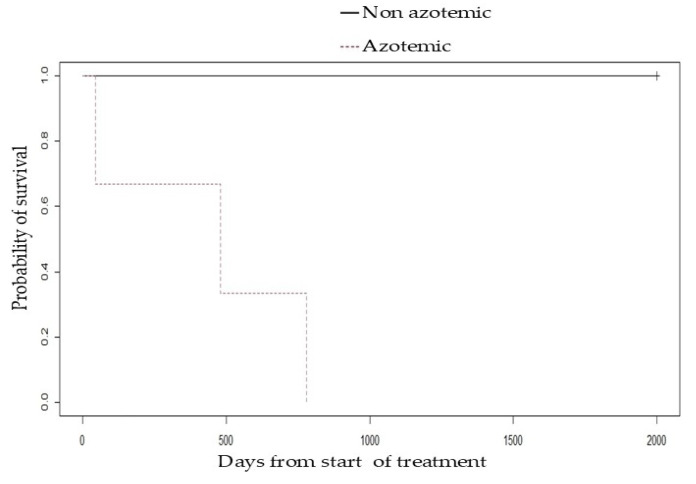
Kaplan–Meier survival curve comparing dogs with and without azotemia at diagnosis; all were treated with chlorambucil for suspected immune-mediated glomerular disease.

**Figure 5:**
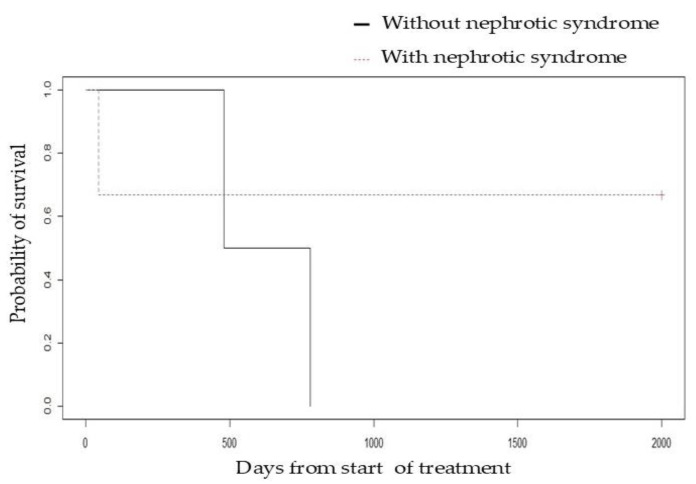
Kaplan–Meier survival curve comparing dogs with and without nephrotic syndrome treated with chlorambucil monotherapy for immune-mediated protein-losing nephropathy.

The overall survival of the patients treated with immunosuppressive therapy ranged from 45 to 2880 days, with a median of 780 days. The euthanasia of one patient during treatment due to poor quality of life as perceived by his or her caregivers, the death of two patients due to progression of CKD, and the death of two patients due to causes unrelated to renal disease were recorded. A lower survival rate was observed in azotemic patients (Figure 4) and in those with hyperphosphatemia (Figure S2) prior to treatment with chlorambucil, but there were no statistically significant differences between the two groups (P = 0.06). The presence of nephrotic syndrome was not associated with lower survival (Figure 5) (P = 0.4).

Finally, in none of the patients who completed the therapeutic protocol was it necessary to reinstate immunosuppressive therapy after its suspension.

## 4. Discussion

This preliminary study describes the clinical and paraclinical responses to chlorambucil monotherapy in dogs with suspected immune-mediated, protein-losing nephropathy (PLN). All treated dogs showed a sustained reduction in proteinuria and improved serum albumin concentrations, and four dogs showed clinical stabilization, supporting the potential efficacy and safety of this treatment approach.

Protein-losing nephropathy (PLN) in dogs represents a complex clinical entity frequently associated with an immune-mediated glomerular lesion. Within these, immunocomplex-mediated glomerulonephritis (ICGN) is recognized as the most prevalent cause, representing approximately 30–50% of the cases diagnosed with renal biopsy [7,8,9]. In this context, the choice of an adequate and effective immunosuppressive treatment is essential to reduce the sequestration of inflammatory mediators in the glomerular territory, modulate the inflammatory response, reduce renal damage, and consequently improve long-term clinical outcomes. This becomes even more relevant in patients in whom the underlying disease leading to the development of ICGN has not been identified and eliminated. The scarce available evidence does not allow predicting the clinical response to treatment, which makes the choice of immunosuppressive therapy difficult. The present case report documents the clinical and paraclinical response to chlorambucil monotherapy in dogs with clinical suspicion of immunocomplex-mediated PLN, providing preliminary evidence on its possible efficacy and safety. Unlike other immunosuppressants used in PLN, such as mycophenolate mofetil, chlorambucil has not previously been evaluated in isolation as a therapeutic agent in this setting in veterinary medicine, positioning this preliminary case series as a meaningful addition to the existing veterinary evidence on therapeutic approaches to ICGN chlorambucil is an alkylating agent belonging to the group of nitrogenated mustards that has been traditionally used as a chemotherapeutic agent in lymphoproliferative processes, but chlorambucil has also demonstrated usefulness in immunomediated diseases of dermatological origin, such as pemphigus foliaceus, discoid and systemic lupus erythematosus, immune-mediated vasculitis, and cold agglutinin disease, among others [25,26]. The immunosuppressive profile of alkylating agents has justified their use in human medicine in the treatment of mesangioproliferative glomerulonephritis and IgA nephropathy, usually in combination with corticosteroids [20,27]. This therapeutic background in humans rationally supports its exploration in veterinary patients with immune-based glomerulopathies.

In this report, all patients treated with chlorambucil showed a decrease in the urine protein–creatinine ratio (UPC) greater than 50% with respect to the baseline value, and one of them showed a decrease to a value less than 0.5. A decrease in proteinuria greater than 50% is considered a marker of favorable response to treatment [15]. The sustained decrease in UPC, the increase in serum albumin, and the stabilization of creatinine values strongly suggest that chlorambucil was effective in modulating the inflammatory response associated with glomerular damage, limiting renal injury. It is noteworthy that this improvement was achieved without the need to resort to combined regimens with other immunosuppressants, which reduces the potential risk of undesirable side effects.

The median survival time of the dogs was 780 days (range 45 to 2880 days), where therapy was completed in three of five patients. Although a shorter median survival time was observed in azotemic and hyperphosphatemic patients (435 days) compared to non-azotemic patients (2520 days), no statistically significant differences were found between the two groups, which is likely influenced by the small number of dogs included in this study. Future studies involving a larger cohort of dogs are warranted to determine whether the presence of azotemia and hyperphosphatemia at the time of PLN diagnosis is indeed associated with prognosis. The three patients who developed azotemia had serum creatinine concentrations ranging from 1.4 to 2.8 mg/dL (mean 2.3 mg/dL) prior to the initiation of immunosuppressive therapy; two of these patients exhibited a sustained increase in creatinine before starting standard therapy. This increase worsened following the initiation of telmisartan treatment, with both patients showing a greater than 25% increase from their pre-treatment baseline. In these cases, telmisartan was discontinued when immunosuppressive therapy with chlorambucil was initiated. Both dogs subsequently showed a sustained decrease in serum creatinine levels starting 30 days after initiation of treatment, although complete normalization was not achieved. One patient experienced an increase in creatinine of less than 25% (from 1.4 to 1.6 mg/dL) during immunosuppressive therapy. These findings suggest that the use of chlorambucil may help limit active renal injury secondary to the sequestration of inflammatory mediators within the glomerular compartment, thereby promoting the recovery of glomerular function. However, early diagnosis and timely intervention prior to the development of azotemia may be essential for improving patient prognosis.

In contrast to what was reported by Klosterman et al. (2011) [28], our data did not show a negative correlation between the presence of nephrotic syndrome and survival time. In our sample, the median lifespan of patients who developed nephrotic syndrome was 1,815 days, compared to 630 days for those who did not. While this observation could suggest that the development of nephrotic syndrome is not necessarily associated with more severe glomerular damage or a rapid decline in glomerular filtration rate, the limited sample size and statistical power of our study preclude definitive conclusions. Therefore, further studies with a larger cohort of patients are needed to clearly define the influence of nephrotic syndrome on the prognosis of dogs with PLN. The mechanisms involved in the development of nephrotic syndrome are associated with decreased oncotic pressure and increased distal tubular sodium reabsorption due to overstimulation of the renin angiotensin aldosterone system leading to volume retention and hypertension [29]; however, in this study none of the patients who developed nephrotic syndrome showed signs of hypervolemia and hypertension. In turn, the three patients with nephrotic syndrome presented absence of abdominal effusion and/or peripheral edema 48 h after initiating immunosuppressive treatment, but the correction of serum albumin values was achieved 30 days after treatment, suggesting that the decrease in oncotic pressure is not the predominant factor in the formation of edema or abdominal effusion.

Compared with previously published data on the effect of mycophenolate in patients with biopsy-confirmed and immune-mediated glomerulonephritis [18], the use of chlorambucil as immunosuppressive monotherapy in this preliminary study demonstrated a sustained reduction in proteinuria and survival times that are comparable with immunosuppressive monotherapies with mycophenolate or with combination immunosuppressive therapies.

Regarding the underlying etiology, unlike what has been reported in North America or in Europe where the development of ICGN is mainly associated with infectious diseases [30,31,32,33], no systemic, neoplastic, infectious, or chronic non-infectious inflammatory systemic pathology could be identified in four of the patients treated with chlorambucil, raising the possibility of idiopathic ICGN or of primary autoimmune origin. Only one patient was diagnosed with immune-mediated hemolytic anemia and ICGN. The absence of endemic vector-borne diseases in Chile, such as leishmaniasis, borreliosis, and Dirofilaria immitis infection, together with the occurrence of only isolated cases of rickettsial infections [34], may partially account for the low number of dogs identified in this study with suspected ICGN. It is noteworthy that tolerance to chlorambucil treatment was adequate in all patients included, with no significant reports of adverse effects. This is particularly relevant considering that other immunosuppressants, such as mycophenolate and glucocorticoids, present more limited safety profiles, including diarrhea, vomiting, gastrointestinal ulcers, leukopenia, increased susceptibility to opportunistic infections, development of iatrogenic hyperadrenocorticism syndrome [35,36]. The absence of severe side effects in this small group suggests that chlorambucil may represent a safe alternative for the management of ICGN, particularly in patients in whom combination therapy is contraindicated or not well tolerated. However, the use of a cytostatic drug, such as chlorambucil, requires informing dog owners about the potential risks of administering this type of drug at home, as well as giving detailed instructions on how to handle it, stressing the need to use disposable gloves, avoid splitting or crushing tablets, handle organic waste with gloves, and dispose of it in sealed bags, as well as preventing pregnant women and children from handling the drug.

In this report there was no evidence of thromboembolism in any of the dogs included in the study. However, although the Consensus Recommendations for Standard Therapy of Glomerular Disease in Dogs [15] recommends the use of aspirin as antithrombotic prophylaxis in dogs, it is currently recognized that aspirin should not be the first choice, given its lower safety profile and the limited and conflicting evidence available on its efficacy, so the use of low molecular weight heparins or combined therapy of clopidogrel and low molecular weight heparins should be favored when faced with a high risk of venous thrombosis [37].

On the other hand, this report included two predisposed breeds (Cocker Spaniels and Mastiffs) to developing primary glomerulopathies, such as hereditary nephritis. This condition typically presents between 9 and 15 months of age and is caused by a defect in type IV collagen within the glomerular basement membrane [38,39]. Its primary clinical manifestation is proteinuria; therefore, performing a renal biopsy in young patients of these breeds is particularly important to establish a differential diagnosis and to guide appropriate pharmacological therapy. However, based on the clinical characteristics and the age at presentation of the patients included in this study, hereditary nephritis was ruled out as a presumptive diagnosis.

The main limitations of this study include its retrospective nature, the small sample size, the absence of histopathologic confirmation of glomerular lesions and the use of different non-standardized standard therapy in conjunction with immunosuppressive therapy with chlorambucil. While clinical and laboratory findings were consistent with ICGN, definitive diagnosis would require renal biopsy and immunohistochemistry. Additionally, the lack of a control group precludes definitive conclusions regarding the efficacy of chlorambucil relative to other immunosuppressants.

Finally, it is essential to emphasize that the therapeutic approach to glomerular disease in dogs should be individualized, considering not only biochemical and clinical parameters but also comorbid conditions, drug tolerance, and the availability of resources for continuous monitoring. The choice of immunosuppressant should consider not only its efficacy but also its safety profile and the patient’s long-term quality of life.

These preliminary findings warrant further investigation in prospective, controlled clinical trials to validate the therapeutic role of chlorambucil in the management of immune-mediated glomerular disease in dogs.

## 5. Conclusions

The findings presented suggest that chlorambucil can be a useful and well-tolerated therapeutic alternative in dogs with suspected immune complex-mediated PLN, especially in those cases in which the aim is to avoid combination regimens or where there are contraindications to other immunosuppressants. The favorable response observed in parameters such as UPC, serum albumin, and creatinine, together with the absence of significant adverse events, support its inclusion as a therapeutic option to be considered. However, it is essential to continue generating scientific evidence through controlled clinical studies that define more precisely its role in the treatment of immune-mediated glomerular disease in veterinary medicine. Controlled clinical trials are warranted to further evaluate the efficacy, safety, and optimal treatment protocols for chlorambucil in canine immune-mediated glomerular disease.

## Figures and Tables

**Table 1:** Clinical characteristics of five dogs with suspected immune-mediated, protein-losing nephropathy prior to initiation of chlorambucil therapy, including age, presence of nephrotic syndrome (NS), urine protein-to-creatinine ratio (UPC), serum creatinine, albumin, and cholesterol concentrations.

Patient	Age (Years)	NS ^1^	UPC ^2^	Creatinine (mg/dL)	Albumin (mg/dL)	Cholesterol ^3^ (mg/dL)
1	6	Present	6.5	0.6	1.3	422
2	7	Present	13.2	1.4	1.2	395
3	12	Absent	6.6	2.8	2.2	192
4	5	Present	11.8	0.6	1.7	351
5	6	Absent	8.1	2.8	2.4	311

^1^ Nephrotic syndrome; ^2^ Urine protein–creatinine ratio; ^3^ Cholesterol reference interval: 120–275 mg/dL.

**Table 2:** Percent change from baseline in serum creatinine (Δ%Cr), hematocrit (Δ%Hct), leukocytes (Δ%WBC), and platelets (Δ%PLT) at 15, 30, and 60 days after initiating chlorambucil monotherapy in dogs with suspected immune-mediated protein-losing nephropathy.

Variable	Day Treatment	Mean	Sd	n
∆%Cr	15	30.1	37.9	5
	30	−3.2	19.0	5
	60	−4.7	18.0	4
∆%Hct	15	29.5	54.2	5
	30	33.5	61.0	5
	60	62.2	83.5	4
∆%WBC	15	−22.3	20.3	5
	30	−20.8	15.0	5
	60	−31.4	26.0	4
∆%PLT	15	−5.0	64.5	5
	30	0.8	60.5	5
	60	−24.1	31.5	4

## Data Availability

The authors confirm that the data supporting the findings of this study are available within the article.

## Supplementary Materials

The following supporting information can be downloaded at: https://www.mdpi.com/article/10.3390/vetsci12080721/s1. Figure S1a: Longitudinal evaluation of creatinine values for the 5 dogs diagnosed with PLN, treated with chlorambucil; Fiure S1b: Longitudinal evaluation of Leukocytes values for the 5 dogs diagnosed with PLN, treated with chlorambucil; Figure S1c: Longitudinal evaluation of platelets values for the 5 dogs diagnosed with PLN, treated with chlorambucil; Figure S2: Kaplan–Meier survival curve comparing dogs with and without hyperphosphatemia treated with chlorambucil monotherapy for immune-mediated protein-losing nephropathy.

## References

[1] Dadousis C., Whetton A.D., Mwacalimba K., Merlo A., Wright A., Geifman N. (2024). Renal Disease in Cats and Dogs-Lessons Learned from Text-Mined Trends in Humans. Animals.

[2] Pelander L., Ljungvall I., Egenvall A., Syme H., Elliott J., Häggström J. (2015). Incidence of and mortality from kidney disease in over 600,000 insured Swedish dogs. Vet. Rec..

[3] O’Neill D.G., Elliott J., Church D.B., McGreevy P.D., Thomson P.C., Brodbelt D.C. (2013). Chronic kidney disease in dogs in UK veterinary practices: Prevalence, risk factors, and survival. J. Vet. Intern. Med..

[4] Rudinsky A.J., Harjes L.M., Byron J., Chew D.J., Toribio R.E., Langston C., Parker V.J. (2018). Factors associated with survival in dogs with chronic kidney disease. J. Vet. Intern. Med..

[5] Jacob F., Polzin D.J., Osborne C.A., Neaton J.D., Kirk C.A., Allen T.A., Swanson L.L. (2005). Evaluation of the association between initial proteinuria and morbidity rate or death in dogs with naturally occurring chronic renal failure. J. Am. Vet. Med. Assoc..

[6] Macdougall D.F., Cook T., Steward A.P., Cattell V. (1986). Canine chronic renal disease: Prevalence and types of glomerulonephritis in the dog. Kidney Int..

[7] Aresu L., Martini V., Benali S.L., Brovida C., Cianciolo R.E., Dalla Riva R., Trez D., Van Der Lugt J.J., Van Dongen A., Zini E. (2017). European Veterinary Renal Pathology Service: A Survey Over a 7-Year Period (2008–2015). J. Vet. Intern. Med..

[8] Crivellenti L.Z., Silva G.E., Borin-Crivellenti S., Cianciolo R., Adin C.A., Dantas M., Dos Anjos D.S., Tinucci-Costa M., Santana A.E. (2016). Prevalence of Glomerulopathies in Canine Mammary Carcinoma. PLoS ONE.

[9] Schneider S.M., Cianciolo R.E., Nabity M.B., Clubb F.J., Brown C.A., Lees G.E. (2013). Prevalence of immune-complex glomerulonephritides in dogs biopsied for suspected glomerular disease: 501 cases (2007–2012). J. Vet. Intern. Med..

[10] Schultz D.M., Rivera C., Jeffery N., Cianciolo R.E., Hokamp J.A., Labato M.A., Nabity M.B. (2025). Analysis of survival among biopsy-determined categories of kidney disease in dogs. J. Vet. Intern. Med..

[11] Pasca S., Solcan G. (2025). Current data regarding the etiopathogenesis of glomerular and tubular disorders associated with kidney diseases in small animals: A review. Rev. Rom. Med. Vet..

[12] Couser W.G. (2012). Basic and translational concepts of immune-mediated glomerular diseases. J. Am. Soc. Nephrol..

[13] Littman M.P. (2011). Protein-losing nephropathy in small animals. Vet. Clin. N. Am. Small Anim. Pract..

[14] Cianciolo R.E., Mohr F.C., Aresu L., Brown C.A., James C., Jansen J.H., Spangler W.L., van der Lugt J.J., Kass P.H., Brovida C. (2016). World Small Animal Veterinary Association Renal Pathology Initiative: Classification of Glomerular Diseases in Dogs. Vet. Pathol..

[15] Brown S., Elliott J., Francey T., Polzin D., Vaden S. (2013). Consensus recommendations for standard therapy of glomerular disease in dogs. J. Vet. Intern. Med..

[16] Lourenço B.N., Coleman A.E., Brown S.A., Schmiedt C.W., Parkanzky M.C., Creevy K.E. (2020). Efficacy of telmisartan for the treatment of persistent renal proteinuria in dogs: A double-masked, randomized clinical trial. J. Vet. Intern. Med..

[17] Pressler B., Vaden S., Gerber B., Langston C., Polzin D. (2013). Consensus guidelines for immunosuppressive treatment of dogs with glomerular disease absent a pathologic diagnosis. J. Vet. Intern. Med..

[18] Vessieres F., Cianciolo R.E., Gkoka Z.G., Kisielewicz C., Bazelle J., Seth M., Adam F.H., Matiasovic M., Aresu L., Jepson R.E. (2019). Occurrence, management and outcome of immune-complex glomerulonephritis in dogs with suspected glomerulopathy in the UK. J. Small Anim. Pract..

[19] Segev G., Cowgill L.D., Heiene R., Labato M.A., Polzin D.J. (2013). Consensus recommendations for immunosuppressive treatment of dogs with glomerular disease based on established pathology. J. Vet. Intern. Med..

[20] Chiarenza D.S., Verrina E.E., La Porta E., Caridi G., Ghiggeri G.M., Mortari G., Lugani F., Angeletti A., Bigatti C. (2024). Biologics and Non-Biologics Immunosuppressive Treatments for IgA Nephropathy in Both Adults and Children. J. Clin. Med..

[21] Floege J., Amann K. (2016). Primary glomerulonephritides. Lancet.

[22] Du W., Chen Z., Fang Z., Li J., Weng Q., Zheng Q., Xie L., Yu H., Gu X., Shi H. (2023). Oral glucocorticoids with intravenous cyclophosphamide or oral glucocorticoids alone in the treatment of IgA nephropathy present with nephrotic syndrome and mesangioproliferative glomerulonephritis. Clin. Kidney J..

[23] Viviano K.R. (2022). Glucocorticoids, Cyclosporine, Azathioprine, Chlorambucil, and Mycophenolate in Dogs and Cats: Clinical Uses, Pharmacology, and Side Effects. Vet. Clin. N. Am. Small Anim. Pract..

[24] Lai Y.-Y., Horta R.D.S., Valenti P., Giuliano A. (2024). Retrospective Safety Evaluation of Combined Chlorambucil and Toceranib for the Treatment of Different Solid Tumours in Dogs. Animals.

[25] Rosenkrantz W.S. (2004). Pemphigus: Current therapy. Vet. Dermatol..

[26] Miller W.H., Griffin C.E., Campbell K.L., Miller W.H., Griffin C.E., Campbell K.L. (2013). Autoimmune and Immune-Mediated Dermatoses. Muller and Kirk’s Small Animal Dermatology.

[27] Samuels J.A., Strippoli G.F., Craig J.C., Schena F.P., Molony D.A. (2003). Immunosuppressive agents for treating IgA nephropathy. Cochrane Database Syst. Rev..

[28] Klosterman E.S., Moore G.E., de Brito Galvao J.F., DiBartola S.P., Groman R.P., Whittemore J.C., Vaden S.L., Harris T.L., Byron J.K., Dowling S.R. (2011). Comparison of signalment, clinicopathologic findings, histologic diagnosis, and prognosis in dogs with glomerular disease with or without nephrotic syndrome. J. Vet. Intern. Med..

[29] Politano S.A., Colbert G.B., Hamiduzzaman N. (2020). Nephrotic Syndrome. Prim. Care.

[30] Crivellenti L.Z., Cintra C.A., Maia S.R., Silva G.E.B., Borin-Crivellenti S., Cianciolo R., Adin C.A., Tinucci-Costa M., Pennacchi C.S., Santana A.E. (2021). Glomerulotubular pathology in dogs with subclinical ehrlichiosis. PLoS ONE.

[31] Mehrkens L.R., Mohr F.C., Sykes J.E. (2016). Clinicopathologic and Histopathologic Renal Abnormalities in Dogs with Coccidioidomycosis. J. Vet. Intern. Med..

[32] Máthé A., Dobos-Kovács M., Vörös K. (2007). Histological and ultrastructural studies of renal lesions in *Babesia canis* infected dogs treated with imidocarb. Acta Vet. Hung..

[33] Aresu L., Benali S., Ferro S., Vittone V., Gallo E., Brovida C., Castagnaro M. (2013). Light and electron microscopic analysis of consecutive renal biopsy specimens from leishmania-seropositive dogs. Vet. Pathol..

[34] Di Cataldo S., Cevidanes A., Ulloa-Contreras C., Hidalgo-Hermoso E., Gargano V., Sacristán I., Sallaberry-Pincheira N., Penaloza-Madrid D., González-Acuña D., Napolitano C. (2021). Mapping the distribution and risk factors of Anaplasmataceae in wild and domestic canines in Chile and their association with Rhipicephalus sanguineus species complex lineages. Ticks Tick Borne Dis..

[35] Tham H.L., Davis J.L. (2024). Pharmacology of drugs used in autoimmune dermatopathies in cats and dogs: A narrative review. Vet. Dermatol..

[36] Fukushima K., Lappin M., Legare M., Veir J. (2021). A retrospective study of adverse effects of mycophenolate mofetil administration to dogs with immune-mediated disease. J. Vet. Intern. Med..

[37] Goggs R., Bacek L., Bianco D., Koenigshof A., Li R.H. (2019). Consensus on the rational use of antithrombotics in veterinary critical care (CURATIVE): Domain 2—Defining rational therapeutic usage. J. Vet. Emerg. Crit. Care.

[38] Vaden S., Grauer G., Bartges J., Polzin D. (2011). Glomerular disease. Nephrology and Urology of Small Animals.

[39] Lavoué R., Van der Lugt J., Day M., Georges M., Busoni V., Merveille A.-C., Poujade A., Peeters D. (2010). Progressive juvenile glomerulonephropathy in 16 related French Mastiff (Bordeaux) dogs. J. Vet. Intern. Med..

