# Supplementary material for: Chlorambucil Monotherapy in Dogs with Protein-Losing Nephropathy of Probable Immune Origin: A Preliminary Study
Source: Vet Sci. 2025 Jul 31;12(8):721. doi: 10.3390/vetsci12080721 (PMC12390262; doi:10.3390/vetsci12080721)
Supplement: Supplementary file 1 [file vetsci-12-00721-s001.zip › vetsci-3745988-supplementary.pdf]

Supplementary Material

Supplementary Figures S1

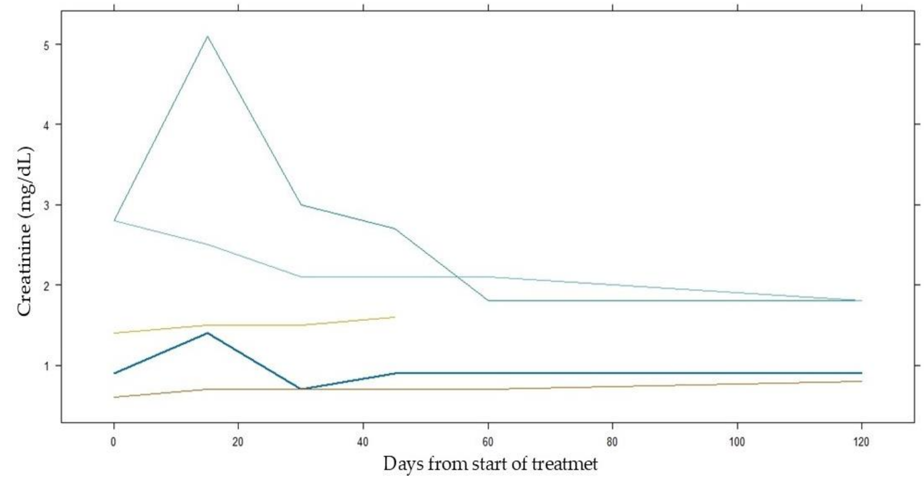

Figure S1a: Longitudinal evaluation of creatinine values for the 5 dogs diagnosed with PLN, treated with chlorambucil.

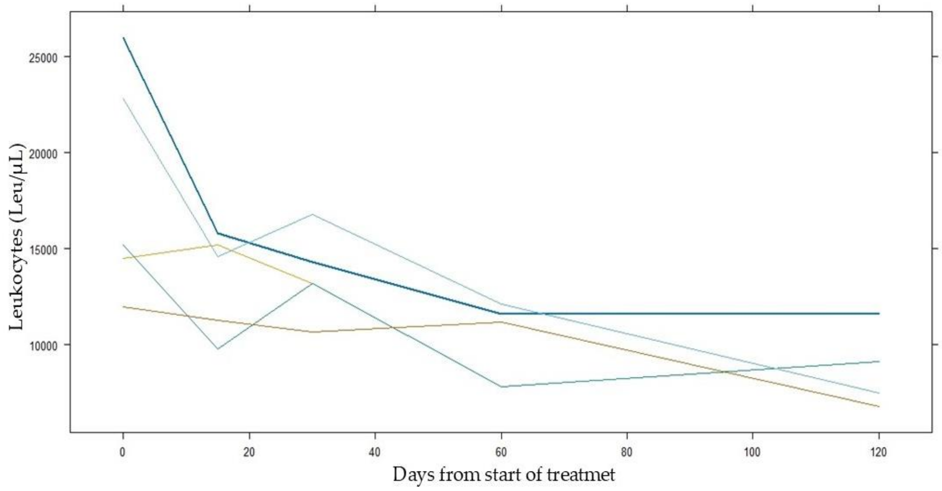

Figure S1b: Longitudinal evaluation of Leukocytes values for the 5 dogs diagnosed with PLN, treated with chlorambucil.

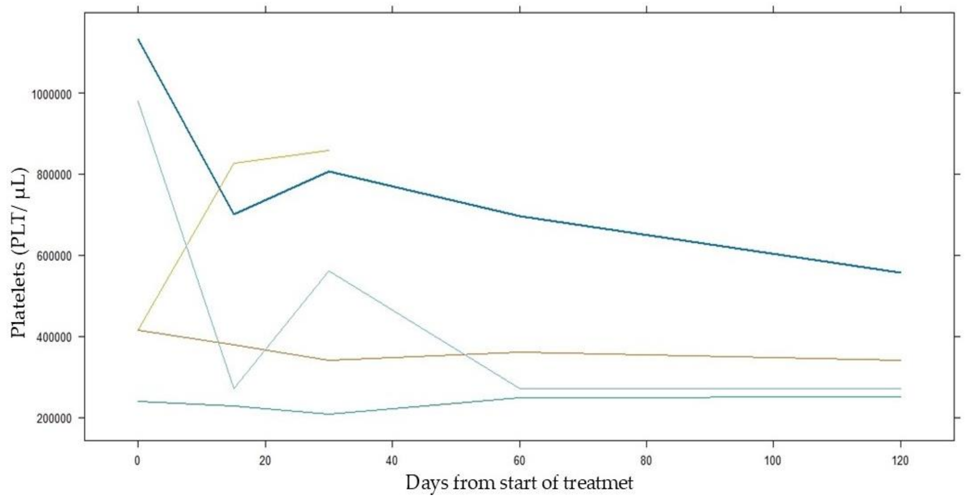

Figure S1c: Longitudinal evaluation of platelets values for the 5 dogs diagnosed with PLN, treated with chlorambucil.

Supplementary Figures S2

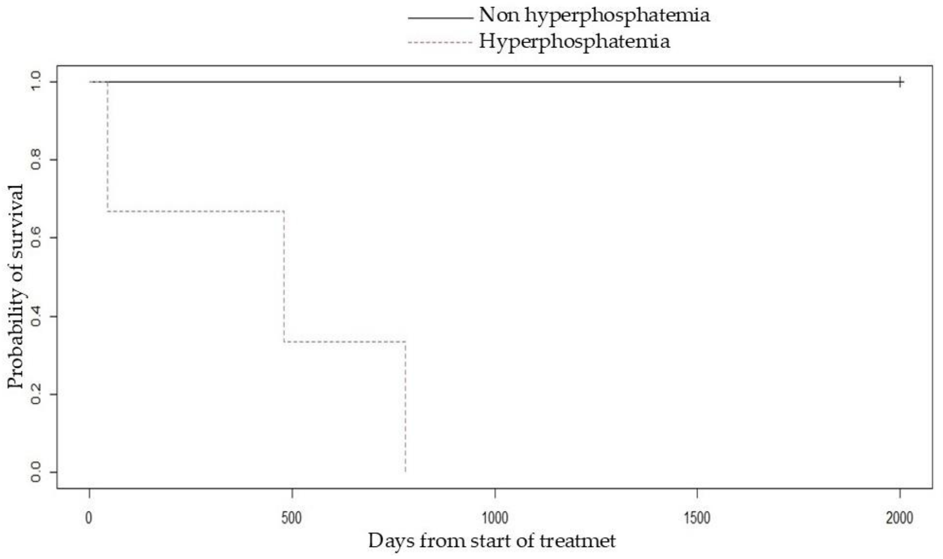

Figure S2: Kaplan–Meier survival curve comparing dogs with and without hyperphosphatemia treated with chlorambucil monotherapy for immune-mediated protein-losing nephropathy.
